# Persistent complete molecular remission after nilotinib and graft-versus-leukemia effect in an acute lymphoblastic leukemia patient with cytogenetic relapse after allogeneic stem cell transplantation

**DOI:** 10.1186/2162-3619-1-29

**Published:** 2012-09-17

**Authors:** Paul Farnsworth, David Ward, Vijay Reddy

**Affiliations:** 1 Florida Hospital Cancer Institute, University of Central Florida, 2501 N. Orange Avenue. Suite 581, Orlando, FL, 32804, USA

**Keywords:** Philadelphia chromosome positive acute lymphoblastic leukemia (PH + ALL), Nilotinib, Extracorporeal photopheresis (ECP), Graft-versus-host disease (GVHD), Graft-versus-leukemia effect (GVL), Hematopoietic stem cell transplantation (HSCT)

## Abstract

We report the successful treatment and sustained molecular remission using single agent nilotinib in a relapsed Philadelphia chromosome positive (Ph+) acute lymphoblastic leukemia patient after allogeneic hematopoietic stem cell transplantation. Compared to previously published studies, this is the first report where a patient did not receive additional chemotherapy after relapse, nor did she receive donor lymphocyte infusions. With nilotinib, the patient reverted back to normal blood counts and 100% donor reconstitution by single tandem repeat (STR) chimerism analysis in the bone marrow and in peripheral blood, granulocytes, T and B-lymphocytes. This report also highlights the use of nilotinib in combination with extracorporeal photopheresis (ECP) for concomitant graft-versus-host disease. Our data suggests that ECP, together with nilotinib, did not adversely affect the overall Graft-versus-leukemia (GVL) effect.

## Background

Philadelphia chromosome (Ph+) is the most common cytogenetic abnormality in adult ALL and is the translocation between chromosome 9 and 22 demonstrating BCR-ABL gene rearrangement and accounts for approximately 20-30% of all adult ALL cases [1]. It carries an adverse prognosis with standard chemotherapy alone, requiring allogeneic bone marrow transplant for a curative approach. First and second generation TKI's were developed to target the BCR-ABL fusion protein [2]. The availability of these new agents is changing the treatment paradigm and the prognosis for these patients. Using imatinib as a single agent showed short-lived responses to treatment, and thus new studies were done using imatinib with concomitant chemotherapy. One such study showed 96% of patients achieving complete remission after a median of 21 days [3]. Second generation TKI's have significantly more potent antileukemia activity against BCR-ABL positive leukemia’s and are under active investigation for ALL [4]. They have also shown great activity in patients with imatinib resistant Ph + ALL. Nilotinib is approximately 30 fold more potent than imatinib and is active in vitro against 32 of 33 BCR/ABL mutations [5]. A phase II study of nilotinib (400 mg twice a day) in relapsed or refractory Ph + ALL reported that 24% of patients attained a complete hematologic response (CHR) [6].

The utility of second generation TKIs as monotherapy in relapsed disease has not yet been fully evaluated [6,7]. We report the successful use of nilotinib as a single agent treatment in obtaining molecular remission for over one year in Ph + ALL that relapsed after allogeneic SCT. This patient reverted back to full donor reconstitution, leading to development of chronic graft-versus-host disease (GVHD). Despite additional immunosuppression and extracorporeal photopheresis (ECP), graft-versus-leukemia (GVL) effect was sustained and the patient remains in molecular remission.

## Case presentation

A 54- year-old woman presented with Philadelphia chromosome-positive precursor B-cell acute lymphoblastic leukemia in March 2009. She received induction chemotherapy consisting of six cycles of Hyper-CVAD chemotherapy regimen. She also received imatinib along with the Hyper-CVAD regimen. A bone marrow biopsy was performed after the sixth cycle, which revealed complete remission (CR). Her central nervous system was negative for disease. The patient then underwent an allogeneic peripheral blood stem cell (PBSC) transplant in November 2009 from her brother who was a 6/6 HLA match with a CD34 cell dose of 7.5 x 10^6^/kg. Our patient was positive for CMV and the donor was CMV negative. The imatinib was stopped three days before her conditioning regimen started. She underwent a reduced intensity-conditioning regimen because of her age and co-morbid conditions, which consisted of hypertension, depression, anxiety disorder, and back pain related to multiple thoracolumbar vertebral compression fractures, due to leukemia, treated with kyphoplasty. The reduced intensity regimen consisted of flubarabine (150 mg/m^2^) and melphalan (140 mg/m^2^). GVHD prophylaxis consisted of FK 506 (Tacrolimus, Prograf) and methotrexate. A bone marrow biopsy done on day 30 showed complete molecular remission with 100% male donor chimerism. However, our patient struggled from ongoing GVHD post-transplant. She developed acute GVHD six weeks after transplant involving the gut stage IV, skin stage III, and liver stage II with an overall acute GVHD of grade IV D. She was treated with high dose corticosteroids (2 mg per kg) but became steroid refractory, requiring the addition of CellCept, rituximab, Entocort, and Rapamune. She responded to this treatment and her immunosuppression was weaned down to 10 mg of Prednisone daily along with cyclosporine. She was in complete molecular remission based RT-PCR and was 100% donor chimerism by single tandem repeat testing for Granulocytes, T-Lymphocytes and B-Lymphocytes and had male XY chromosome cytogenetics in the bone marrow. Eight months after transplant, due to her persistent need for immunosuppression due to her GVHD, she was started on imatinib but at a low dose of 50 mg daily because of thrombocytopenia. Nine months after transplant she was diagnosed with Chronic GVHD involving the mouth, eyes, and skin with increased skin thickening and scleroderma of the lower back and thighs.

One year after HSCT, a bone marrow biopsy was negative for any residual leukemia by both flow cytometry and morphology. Cytogenetics was also normal. However, quantative RT PCR analysis of BCR/ABL gene arrangements for 9; 22 translation showed a positive minor breakpoint of 0.014%. Upon seeing these results, her imatinib was increased from 50 mg to 100 mg daily. She could not tolerate a greater dose of imatinib due to her low platelet count. A subsequent bone marrow biopsy performed in December 2010 showed recurrent acute precursor B-Lymphoblastic leukemia compromising 71% of total cells. Cytogenic analysis also showed that 4 cells of 20 analyzed were female karyotype with deletion 3 and translocation 9; 22. This was new as her previous chromosome analyses following transplant have shown all twenty metaphase cells having a normal karyotype consistent with the donor hematopoiesis and no BCR/ABL cytogenetic translocation was detected in any of the samples. Chimerism studies showed 12% male donor cells, while peripheral blood still remained at 100% donor in granulocytes and T-lymphocytes and B-lymphocytes. In light of this new evidence, the patient expressed her wishes not to undergo aggressive re-induction chemotherapy regimen followed by additional donor lymphocyte infusions. Due to this relapse, in January 2011, fourteen months after her transplant, imatinib was discontinued and nilotinib was started at a dose of 800 mg daily (400 mg p.o.b.i.d.). In February 2011, another biopsy was performed and the morphology by flow cytometry was much improved, but residual acute lymphoblastic leukemia was detected, with approximately 4.4% CD34+ TDT + blasts. A subsequent bone marrow analysis performed two months later in April 2011 showed no evidence of residual disease with RT-PCR being negative. Clinical course for the patient is shown in Table 1.

**Table 1:** Clinical Course of Patient

**Dates (Time from transplant)**	**March 2009 At Diagnosis**	**February 2010 (Day 100)**	**September 2010 (10 Months)**	**October 2010 (11 Months)**	**December 2010 -January 2011 (14 Months)**	**February 2011 (15 Months)**	**April 2011 (17 Months)**	**June 2011- October 2011 (19–24 Months)**
Bone Marrow Biopsy Morphology	Hypercellular 90% with 56% B-Lymphoblasts	No evidence of disease	Not done	No evidence of disease	Recurrent disease comprising 71% of total cells	Residual disease 4.4% CD34+ TDT + Blasts	No evidence of residual/recurrent disease	No evidence of residual/ recurrent disease
Donor Chimerism BM/PB	N/A Female recipient	100% male donor by chromosomes and STR (BM)	Not done	100% male donor	12% male donor	100% male donor	Not done	100% male donor
FISH/ PCR BCR/ABL Blood or Bone Marrow	Philadelphia Chromosome Positive	Negative for BCR/ABL translocation by RT-PCR (BM)	Negative for BCR/ABL translocation (PB)	Positive minor breakpoint .014%	FISH positive BCR/ABL; RT PCR positive for the minor breakpoint region 39.22%	4.4 percent by FISH analysis	Negative for BCR/ABL translocation by RT-PCR	Negative for BCR/ABL translocation
TKI treatment	N/A	None	Gleevec 100 mg*	Gleevec 100 mg*	Nilotinib (400 mg po bid started)	Nilotinib (400 mg po bid)	Nilotinib 800 mg (400 mg po bid)	Nilotinib (400 mg po bid)
GVHD	N/A	skin and gut	skin	skin	skin	skin, eyes, mouth, and gut	skin, eyes, mouth, and gut	skin, eyes, mouth, and gut
ECP	N/A	None	None	None	None	None	None	ECP **
IST	N/A	Cyclosporine and Prednisone	Cyclosporine and Prednisone	Weaned	Discontinued	Cyclosporine: 125 mg p.o.b.i.d.	Cyclosporine: 150 mg p.o.b.i.d.	CellCept: 1000 mg p.o.b.i.d.
						Prednisone: 25 mg daily		Prednisone: 10 mg

* The patient was unable to tolerate a higher dose of Gleevec due to low platelet counts.

**ECP treatments- Treatments were scheduled bi weekly for eight weeks, then once every two weeks for eight weeks, then once a month.

*BM* bone marrow.

*PB* peripheral blood.

*FISH* fluorescent in situ hybridization.

*PCR* polymerase chain reaction; *RT-PCR* reverse transcription polymerase chain reaction.

*TKI* tyrosine-kinase inhibitor.

*GVHD* graft-versus-host disease.

*ECP* extracorporeal photopheresis.

*IST* immunosuppressive therapy.

**Figure 1:**
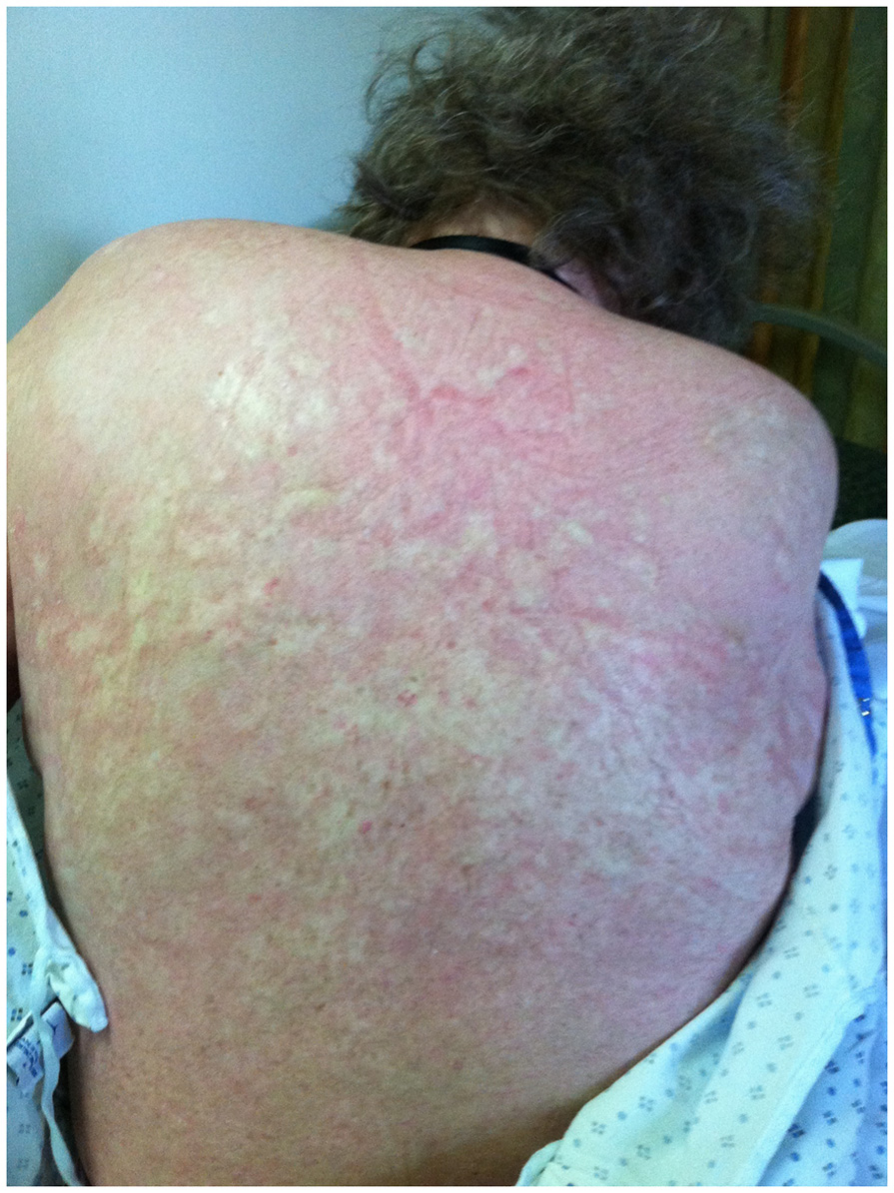
Chronic GVHD of skin.

Her GVHD symptoms continued to worsen after achieving 100% donor engraftment for the second time, and she developed sub-mandibular swelling with acute parotitis. She was then treated with high dose corticosteroids (1mg/kg). In order to reduce steroid toxicity the patient received ECP treatments starting in June 2011 and ending in September 2011, which led to a great improvement of her GVHD symptoms. In August she completed her seventh cycle of ECP treatment, and she stated a great improvement of her mouth and eyesight; she also continued on prednisone 15 mg and cyclosporine 150 mg p.o.b.i.d. However, in September she developed proximal muscle weakness as a form of steroid myopathy. Her steroids were weaned, and CellCept was added because of steroid intolerance. In October, she developed acute renal failure with a creatinine level around 6, and the cyclosporine was stopped as a result. Another biopsy performed in October 2011 showed no evidence of acute lymphoblastic leukemia. Since October 2011, she has been maintained on CellCept, low dose prednisone, and intermittent ECP treatments for her GVHD. Her GVHD is currently stable with skin thickening, sclerodermatous of her legs and upper thighs, eye dryness, and dry mouth (Figure 1). Nilotinib dose is maintained at 800 mg/day and no toxicities were noted requiring dose adjustment or modification. Also, she is currently stable from the leukemia standpoint, with her last biopsy being negative for residual disease, and chimerism studies showed 100% donor cells. Also, her blood counts have maintained a normal range

## Discussion and Conclusion

With the advent of tyrosine kinase inhibitors (TKI), the addition of imatinib to standard therapy has significantly improved outcomes in Ph + ALL [2,8]. However, the use of single agent imatinib has produced temporary response, requiring combination therapies including allogeneic transplantation [9]. In those patients resistant to imatinib, second generation TKIs, such as dasatinib and nilotinib are showing promise in clinical trials [10,11]. We report a combination treatment of single agent nilotinib for relapsed Ph + ALL along with immunosuppressive therapy for GVHD with ECP after related allogeneic stem cell transplantation. Our patient was originally treated with a combination regimen of Hyper-CVAD therapy with concomitant imatinib. After allogeneic transplant imatinib was reinstituted albeit at a lower dose due to thrombocytopenia. Since our patient was already on imatinib and had relapsed, we chose to use nilotinib due to the above-mentioned increased potency of nilotinib and potential to overcome imatinib resistance.

Unlike previous reports, our patient did not receive additional chemotherapy and/or donor lymphocyte infusions after relapse and went into molecular remission on single agent nilotinib, despite 70% blasts in a hyper-cellular marrow [12,13]. One month after starting nilotinib she reverted back to 100% donor reconstitution, without additional stem cells or lymphocytes. However, this led to progression of her graft-versus-host disease. This had chronic features and the patient was steroid intolerant, due to steroid myopathy and weakness. Therefore, ECP was considered a suitable choice, especially considering her sclerodermatous changes. There is promising data about using ECP in steroid resistant/intolerant GVHD [14-16]. We were concerned about this additional immunosuppression to treat her GVHD, since this may abrogate her GVL effect. However, despite the use of corticosteroids, cyclosporine, CellCept, and ECP the patient remains in molecular remission with single agent nilotinib.

ECP is currently FDA approved for the treatment of Cutaneous T-Cell lymphoma (CTCL) and is a well-known treatment option for steroid-refractory chronic graft versus host disease [15-17]. The mechanism of action is not well studied. However, recent data suggests that it is possible to reduce GVHD without generalized immunosuppression [18]. ECP can affect alloreactive T-Cell activity, dendritic cells, and monocytes [19]. A recent murine study demonstrated that ECP attenuates GVHD but preserves dendritic cell vaccination response [20]. Overall these studies suggest that unlike conventional immunosuppression, ECP may selectively target the immune system and separate GVHD from GVL. Our patient could not tolerate higher doses of conventional immunosuppression due to steroid myopathy and nephrotoxicity from cyclosporine. However, her GVHD improved on ECP, and she was able to continue nilotinib to maintain remission.

In conclusion, molecular remission and graft-versus-leukemia effect in Ph + ALL is sustained with the use of nilotinib as a single agent without additional chemotherapy or DLI.

## Consent

Institutional review board informed consent was obtained from the patient for collection of data and for research purposes.

## Competing interests

Authors state that they have no competing interests.

## Author's contributions

PF and VR collected and reviewed data to compile into the paper. DW provided evidence and supporting data. All the authors reviewed and approved the final version of the report.
